# Characterization of Treatment-Naive HIV/HBV Co-Infected Patients Attending ART Clinic of a Tertiary Healthcare Centre in Eastern India

**DOI:** 10.1371/journal.pone.0073613

**Published:** 2013-08-30

**Authors:** Debraj Saha, Ananya Pal, Avik Biswas, Rajesh Panigrahi, Neelakshi Sarkar, Jayeeta Sarkar, Manisha Pal, Subhasish Kamal Guha, Bibhuti Saha, Sekhar Chakrabarti, Runu Chakravarty

**Affiliations:** 1 ICMR Virus Unit, Kolkata, ID & BG Hospital Campus, Kolkata, India; 2 Calcutta School of Tropical Medicine, Kolkata, India; 3 National Institute of Cholera and Enteric Diseases, Kolkata, India; 4 Department of Statistics, University of Calcutta, Kolkata, India; Saint Louis University, United States of America

## Abstract

**Objective:**

The study was designed to assess the hepatitis B virus (HBV) and hepatitis C virus (HCV) co-infection scenario among the human immunodeficiency virus (HIV) infected patients attending a tertiary healthcare unit in eastern India. Additionally, clinical and virological characterization of these viruses, prior to antiretroviral therapy (ART) initiation was also done for better understanding of the disease profile.

**Methods:**

Pool of ART-naive HIV/HBV co-infected and HIV mono-infected patients, participating in two different studies, were included in this study. HBV DNA was detected by nested-PCR amplification followed by HBV genotype determination and HBV reverse transcriptase (RT) region amplification and direct sequencing for detecting drug resistance.

**Results:**

The prevalence of HBsAg (11.3%) was higher compared to anti-HCV (1.9%) among the HIV infected ART-naive patients. Moreover, majority of the HBeAg positive HIV/HBV co-infected patients (87.7%) had HBV DNA ≥20,000 IU/ml with median HBV DNA significantly higher than that of HBeAg negative subjects (5.7 log_10_ IU/ml vs. 4.2 log_10_ IU/ml; p<0.0001). Multivariate analysis also showed that HBeAg-positive status was independently associated with higher HBV DNA level (p = <0.001). Notably, 60.9% of the HBeAg negative co-infected subjects had HBV DNA ≥2,000 IU/ml of which 37.0% had HBV DNA ≥20,000 IU/ml. Genotype HBV/D (68.2%) was the predominant genotype followed by HBV/A (24.3%) and HBV/C (7.5%). Anti-HBV drug resistant mutations were detected in two (3.8%) of the ART-naive patients.

**Conclusion:**

The prevalence of HIV/HBV co-infection was relatively higher in our study subjects. HBeAg testing might provide clue for early treatment initiation. Furthermore, HBeAg negative patients are also associated with high HBV DNA levels and therefore require appropriate medical attention. Pre-treatment screening for anti-HBV drug resistant mutations is not necessary before ART initiation.

## Introduction

Human immunodeficiency virus (HIV) infection is a global health problem affecting approximately 40 million people worldwide [1]. HIV shares its routes of transmission with hepatitis B virus (HBV) and hepatitis C virus (HCV) and therefore co-infection with these viruses is a common phenomenon [2], [3].

Ever since the introduction of antiretroviral therapy (ART), liver disease has turned out to be the second most relevant cause of mortality among the HIV infected individuals worldwide [4]. In patients co-infected with HIV and HBV or HCV, liver fibrosis rates are accelerated leading to faster progression to end-stage liver disease (ESLD) [5]. Hence, characterization of HBV and HCV co-infection among the HIV infected population is one of the primary foci of the current era. Moreover, in a resource-poor country like India, such characterization will bring about better understanding of the disease profile and help in developing efficient management strategies to control chronic hepatitis among the HIV infected patients.

India has the third largest population living with HIV/Acquired immunodeficiency syndrome (AIDS). The adult HIV prevalence is estimated at 0.28–0.30% in the eastern part of the country [6]. In spite of that very little is known about the prevalence of HBV or HCV co-infection among them. Therefore, the present study investigated the burden of HBV and HCV co-infection among the HIV infected patients seen at a tertiary healthcare centre in eastern India. Furthermore, characterization of hepatitis virus co-infection among the HIV infected patients was also done, prior to ART initiation.

## Methods

### Ethics Statement

This work was a part of the study approved by “The Institutional Ethical Committee, National Institute of Cholera and Enteric Diseases (ICMR)”. Written informed consent was obtained from all the study participants in their native language.

### Study Subjects

The HIV infected participants visiting the ART centre of Calcutta School of Tropical Medicine (Kolkata), were recruited for this study. A total of 1020 HIV infected patients were screened during a span of two years from October 2010 to September 2012. Furthermore, additional 83 individuals co-infected with HIV and HBV who were treatment naive, were also included from another clinical trial (IRIS ID No. 2009-05630), for characterizing HIV/HBV co-infection on a broader spectrum. All the participants of the above clinical trial were positive for hepatitis B surface antigen (HBsAg) at the entry level. Based on a detailed examination, data regarding age, sex, history of alcoholism and possible modes of transmission were obtained.

### Serological Testing

HBV specific enzyme-linked immunosorbent assay (ELISA) kits were used for the detection of HBsAg, HBeAg and Anti-HBe (Diasorin, S.P.A, Saluggia, Italy). Anti-HIV and anti-HCV were tested using ELISA kits from General Biologicals, Taiwan and Bio-Rad, France respectively. All the serological assays were performed according to manufacturer’s instruction.

### DNA Isolation, HBV DNA Detection, HBV Genotyping, Sequencing and Quantification

DNA was extracted from HBsAg positive samples using QIAamp DNA Blood Kit (Qiagen, Hilden, Germany) according to manufacturer’s protocol. HBV DNA was detected by a method described earlier [7]. The HBV DNA was quantified as earlier described [8] and stringent precautions were taken to avoid cross contamination [9].

HBV genotype was determined by polymerase chain reaction-restriction fragment length polymorphism (PCR-RFLP) method, as previously described [10]. The results of PCR-RFLP were further confirmed by means of direct sequencing with Prism Big Dye kit and ABI 3130×l Genetic Analyzer (Applied Biosystems, Foster City, USA).

In order to detect the HBV drug resistant mutations, partial HBV reverse transcriptase (RT) region was amplified by means of nested PCR followed by direct sequencing. The complete protocol and the thermal profile were described previously [11]. The accession numbers for the HBV partial polymerase gene sequenced in this study was deposited in the DDBJ/EMBL/GenBank as KC538884 - KC538898; JX244156 - JX244165; JX244167, JX244168, JX244170, JX244171, JQ349119, JQ349121– JQ349123; JQ349125, JQ349127 - JQ349145.

### RNA Extraction, cDNA Synthesis and HCV RNA Detection

RNA was extracted from 140 µl of plasma of anti-HCV positive samples using QIAamp Viral RNA Mini Kit (Qiagen, Hilden, Germany) according to the manufacturer’s instructions. Extracted RNA was reverse transcribed to corresponding cDNA by using the Revert Aid Reverse Transcriptase Kit (Fermentas, EU). HCV RNA was detected by means of nested PCR by a method mentioned elsewhere [12].

### Statistical Analysis

Data entries, calculation of prevalence and determining the median of different parameters were done using Microsoft Excel. Statistical analysis of the data was performed using MINITAB™ Statistical Software (version: 13.31, Minitab Inc. PA, USA). Mann-Whitney test was done to test the difference in medians and chi-square or Fischer’s exact test was used for comparing categorical data using StatCalc (EpiInfo version 6.0, Centers for Disease Control and Prevention and World Health Organization, Geneva, Switzerland). A multivariable linear regression analysis was also performed to examine the dependence of HBV viral load on age, sex and different HBV characteristics. In all the analyses, p-values <0.05 were considered as statistically significant.

## Results

### Baseline Characteristics of the Study Subjects

Out of 1020 HIV sero-reactive participants, 320 were ART naive and were thus included to evaluate the prevalence of HIV/HBV and HIV/HCV co-infection. Among these 320 study subjects, 216 were males and 104 were females (M:F ∼ 2.08∶1). These patients aged between 18 to 65 years with a median age of 35 years. Of them, 36 (11.3%) were found to be HBsAg positive whereas only 6 (1.9%) HIV infected individuals were anti-HCV positive. The major baseline characteristics of the HIV/HBV co-infected, HIV/HCV co-infected and the HIV mono-infected subjects are presented in Table 1. HCV RNA could be tested in only 4 samples and it was undetectable in all of them. HIV/HBV/HCV triple infection was not found in any of the patients.

**Table 1:** Baseline characteristics of HIV/HBV co-infected (HIV^+^/HBsAg^+^), HIV mono-infected (HIV^+^) and HIV^+^/anti-HCV^+^ patients.

Variable	Total ART-naive patients	HIV^+^	HIV^+^/HBsAg^+^	HIV^+^/anti-HCV^+^	p-value
Patients, n (%)	320	278 (86.8)	36 (11.3)	6 (1.9)	<0.0001*
Age, years, median (IQR)	35 (18–65)	35 (18–65)	37 (21–60)	43.5 (25–53)	0.5#
Male sex, n (%)	216 (67.5)	186 (65.5)	30 (83.3)	5 (83.3)	0.03#
CD4+ T-cell count, cells/mm^3^,median (IQR)	175 (2–1175)	177 (2–1175)	162 (16–903)	117 (20–310)	0.45#
ALT, U/L, median (IQR)	28 (3–250)	26 (3–250)	36 (15–182)	31.5 (17–52)	0.002#
HBV DNA Positive, n (%)	–	–	28 (77.8)	–	

# p-value for comparison between HIV/HBV co-infected and the HIV mono-infected subjects.

* p-value for comparison between HIV/HBV co-infected and the HIV^+^/anti-HCV^+^ subjects.

There was significantly higher predominance of males (83.3% vs. 65.5%; p = 0.03) both among the HIV/HBV and HIV/HCV co-infected population compared to the HIV mono-infected subjects. The median CD4+ T-cell count was relatively lower for both the HIV/HBV (162 cells/mm^3^, inter-quartile range [IQR] 16–903 cells/mm^3^) and the HIV/HCV (117 cells/mm^3^, IQR 20–310 cells/mm^3^) co-infected patients compared to the HIV mono-infected subjects (177 cells/mm^3^, IQR 2–1175 cells/mm^3^) but it was not statistically significant (p = 0.45 & p = 0.35 respectively). However, the HIV/HBV co-infected patients exhibited significantly higher ALT levels (36 U/L, IQR 15–182 U/L vs. 26 U/L, IQR 3–250 U/L; p = 0.002) compared to the HIV mono-infected population (Table 1).

### Characterization of HBV Infection among HIV/HBV Co-infected Patients

In order to characterize the HIV/HBV co-infection, additional 83 individuals co-infected with HIV and HBV who were treatment naive were further included in the study, along with the above 36 HBsAg positive subjects, to form an overall pool of 119 HIV/HBV co-infected patients. Of these 119 subjects, 73 (61.3%) were HBeAg positive while 46 (38.7%) were HBeAg negative. Moreover, 37 (80.4%) of the HBeAg negative patients were anti-HBe positive while the rest were anti-HBe negative. Baseline characteristics of HBeAg positive and HBeAg negative patients are summarized in Table 2.

**Table 2:** Comparison of baseline parameters between HBeAg positive and negative patients among the total 119 HBsAg positive patients.

Variable	Total HBsAg^+^ patients	HBsAg ^+^/HBeAg^+^	HBsAg^+^/HBeAg^−^	p-value*
Patients, n (%)	119	73 (61.3)	46 (38.7)	
Age, years, median (IQR)	35 (21–62)	35 (21–60)	35 (23–62)	0.57
Male sex, n (%)	104 (87.4)	65 (89.0)	39 (84.8)	0.5
CD4+ T-cell count, cells/mm^3^, median (IQR)	198 (16–903)	191 (18–838)	203 (16–903)	0.92
ALT, U/L, median (IQR)	44 (12–406)	43.5 (15–290)	44 (12–406)	0.35
HBV DNA, log_10_ IU/ml, median (IQR)	5.2 (1.84–7.65)	5.7 (2.00–7.65)	4.2 1.84–6.97)	<0.0001
HBV genotype, n (%)	N = 107#	N = 72	N = 35	
A	26 (24.3)	20 (27.8)	6 (17.1)	0.23
C	8 (7.5)	5 (6.9)	3 (8.6)	0.71
D	73 (68.2)	47 (65.3)	26 (74.3)	0.35

* p-value for comparison between HBeAg positive and negative subjects (using Mann-Witney test).

# HBV genotype could be amplified for 107 HIV/HBV co-infected subjects.

Out of these 119 HBsAg positive participants, 108 had detectable HBV DNA in their plasma. HBV genotype could be determined for 107 subjects with one displaying atypical banding patterns in RFLP.

**Figure 1:**
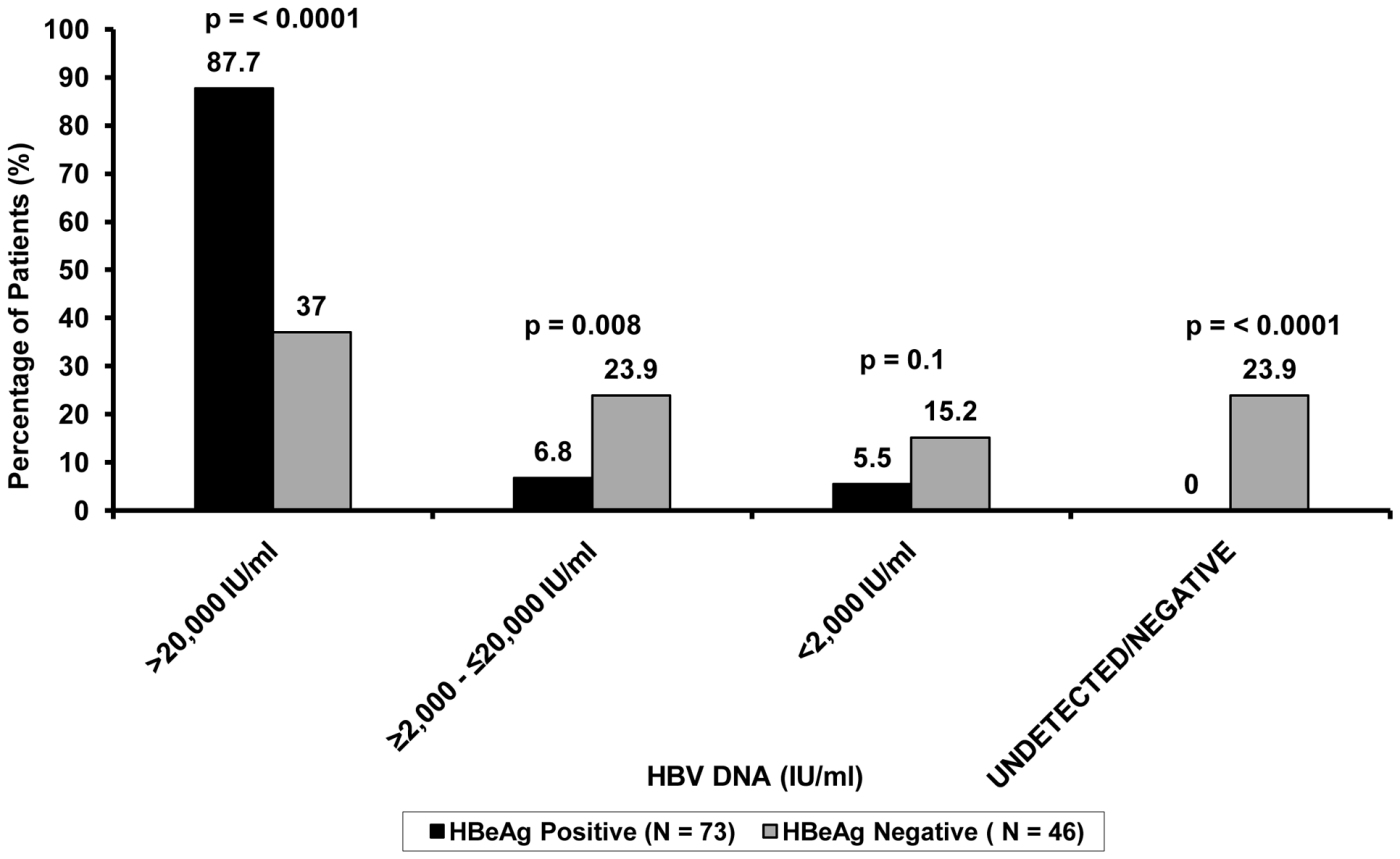
Comparison between HBeAg positive and HBeAg negative subjects with respect to varying HBV DNA levels. Major proportion of HBeAg positive subjects were associated with higher HBV DNA levels compared to HBeAg negative subjects.

Majority of the HBeAg positive patients had HBV DNA ≥20,000 IU/ml (n = 64 [87.7%] vs. n = 17 [37.0%]; p<0.0001) whereas only 12.3% of them had values <20,000 IU/ml (Figure 1). Multivariate analysis also indicated the significant association of HBeAg-positive status with higher HBV DNA load (p-value <0.001, coeff. = 1.364).

On the other hand, 11 (23.9%) patients with HBeAg negative status had undetectable HBV DNA whereas none of the HBeAg positive subjects showed HBV DNA negativity. The proportion of HBeAg negative patients with HBV DNA <2000 IU/ml was only 15.2%. Notably, 28 (60.9%) HBeAg negative co-infected subjects had HBV DNA ≥2,000 IU/ml (Figure 1).

No such significant difference was observed between anti-HBe positive and negative subjects in terms of median HBV DNA load (3.9 log_10_ IU/ml, IQR 1.84–6.97 vs. 3.5 log_10_ IU/ml, IQR 3.26–4.45; p = 0.18).

Furthermore, the partial HBV RT region was sequenced for 53 individuals who were ART naive and were co-infected with HIV and HBV. Only two patients (3.8%) showed the rtM204V and rtL180 M resistant mutations.

### Association of CD4+ T-cell Count with HBV Viremia and HBeAg Status

Since lower CD4+ T-cell count was associated with HIV/HBV co-infection, we further analyzed the variation of CD4+ T-cell count with HBV DNA load among the HBeAg positive and HBeAg negative patients. For this, the CD4+ T-cell count was stratified into two groups of <200 cells/mm^3^ (group 1) and ≥200 cells/mm^3^ (group 2). Of the 73 HBeAg positive participants, CD4+ T-cell count data was available for 71 subjects who were further distributed between group 1 and group 2 containing 37 and 34 patients respectively. Likewise, out of 46 HBeAg negative patients, 22 belonged to group 1 and the rest to group 2.

The median HBV DNA was relatively higher when CD4+ T-cell count was <200 cells/mm^3^ both in case of HBeAg positive and HBeAg negative subjects but the difference was statistically insignificant (p = 0.068 & p = 0.095) (Table 3). However, intergroup comparison between HBeAg positive and negative subjects showed that median HBV DNA was significantly high for HBeAg positive patients both in group 1 and group 2 (p = 0.0002 & p = 0.0001) compared to HBeAg negative patients.

**Table 3:** Variation of HBV DNA levels with CD4+ T-cell count and HBeAg status.

	HBV DNA, log_10_ IU/ml, median (IQR)		p-value^a^
	HBeAg Positive N = 71*	HBeAg Negative N = 35#	
CD4+ T-cell count (<200 cells/mm^3^)Group-1	**n = 37**	**n = 18**	0.0002
	5.9 (2.00–7.65)	4.7 (1.84–6.97)	
CD4+ T-cell count (≥200 cells/mm^3^)Group-2	**n = 34**	**n = 17**	0.0001
	5.6 (2.81–7.56)	3.9 (2.01–6.34)	
**p-value** b	0.068	0.095	

# Only HBV DNA positive subjects were considered from a total of 46 HBeAg negative subjects.

* From a total 73 HBeAg positive subjects, CD4+ T-cell count data was available for 71 subjects based on which analysis was done.

a p-value for inter-group comparison between HBeAg positive and negative subjects with respect to CD4+ T-cell count grouping.

b p-value for intra-group comparison between HBeAg positive and negative patients with CD4+ T-cell count of <200 cells/mm^3^ and ≥200 cells/mm^3.^

**Figure 2:**
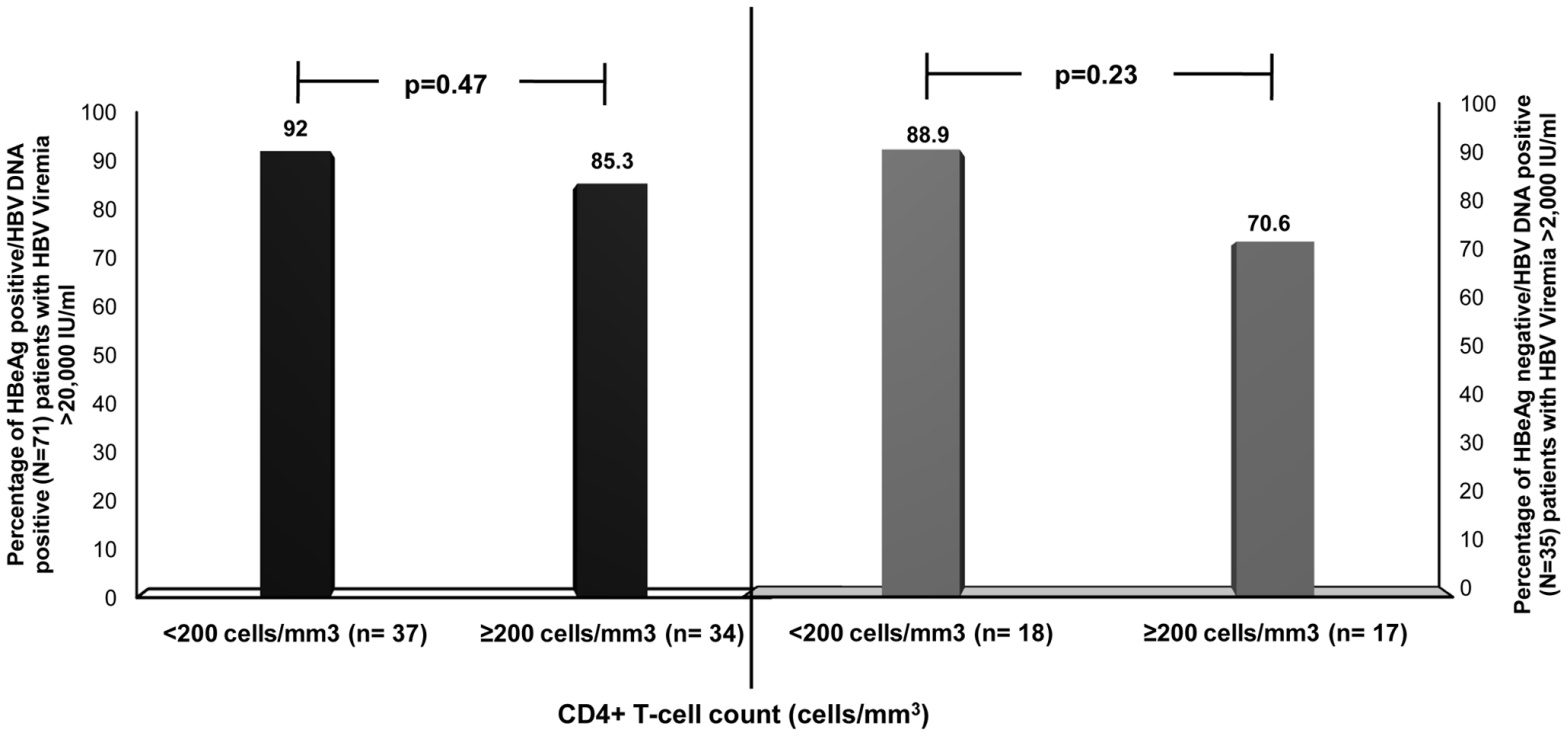
Frequency of subjects with HBV DNA ≥20,000 IU/ml (HBeAg^+^) and ≥2,000 IU/ml (HBeAg^−^) by CD4+ T-cell count. There was no significant difference in the percentage of HBeAg positive patients with HBV DNA ≥20,000 IU/ml between the two CD4+ T-cell count categories (p = 0.47). This indicates the higher association of HBeAg positive subjects with greater HBV viremia, irrespective of CD4+ T-cell count. Similarly, for the HBeAg negative patients, no distinct variation was observed (p = 0.23).

In addition, percentage of HBeAg positive patients with HBV DNA ≥20,000 IU/ml, did not vary significantly between the two groups (p = 0.47). Similar trend was also observed for the HBeAg negative patients with HBV DNA ≥2,000 IU/ml (p = 0.23) (Figure 2).

## Discussion

The present study highlights the predominance of HBV exposure rather than HCV among the HIV infected patients in our setting. Moreover, the presence of high HBV DNA levels in a significant proportion of HBeAg negative patients and the predominance of HBV/D were considered to be the salient findings of our investigation. To the best of our knowledge, this study is the first in India to characterize the hepatitis infection among the HIV infected individuals, at the time of ART initiation.

In our study population, we found that the prevalence of HBsAg (11.3%) was much higher than that of anti-HCV (1.9%) with an overall male predominance. Interestingly, in the general population of India, the prevalence of HBV carriers ranges only between 2%–4% [13]. A possible reason for such marked differences in the prevalence of HBsAg between the two cohorts might be due to the fact that the population under study is HIV positive and thus falls into the high risk group of acquiring HBV infection. Given that the transmission of HCV occurs more efficaciously via percutaneous routes [1], [14], the rate of HCV infection was more frequent among the injection drug users (IDUs). Previous report among the IDUs of Manipur showed higher prevalence of HCV co-infection in HIV infected patients [15]. Since the proportion of IDUs was relatively higher in the north east of India [16], [17], the HCV co-infection was more prevalent there as compared to the eastern part of the country. On the other hand, HBV infection is mostly transmitted by means of sexual routes or perinatally [1]. The fact that male patients are more prone towards acquiring such co-infection, mainly attributable to their higher rate of sexual promiscuity [11], [18], indicates that co-infection with HBV is the major threat among the HIV infected population of eastern India compared to HCV.

As per previous studies from India, which mainly focused on HBV mono-infection, HBV/D was the predominant genotype circulating among the Indian population [19], [20], [21], [22]. Similarly, we found the preponderance of HBV/D followed by HBV/A in our HIV/HBV co-infected patients. Additionally, HBV/C was also found among these co-infected subjects but with a lower frequency. This was consistent with one of the recent findings on HIV/HBV co-infection from this region [11].

The present study also depicts the association of lower CD4+ T-cell count with HIV/HBV co-infection, which is in accordance with one of the first such studies from Nigeria [23]. However, this association was not statistically significant in our settings. Moreover, several studies have suggested that CD4+ T-cell count <200 cells/mm^3^ is often associated with higher HBV DNA levels [23], [24], [25]. Analogous to these observations, our study too showed an association between subjects with CD4+ T-cell count <200 cells/mm^3^ and higher HBV DNA levels, irrespective of HBeAg status (Table 3). However, this was not statistically significant. Recently, a multi-national study by Thio et al, showed that HBeAg positive status was a major factor to be associated with higher HBV DNA levels [26]. Likewise, our study too exemplifies such association between HBeAg positivity and higher HBV DNA load. Furthermore, the proportion of HBeAg positive patients with HBV DNA levels of ≥20,000 IU/ml, the cut-off level recommended for treatment of HBeAg-positive chronic HBV patients [27], did not vary considerably with CD4+ T-cell count (p = 0.47) (Figure 2).

Since HBeAg negative chronic HBV infections are quite frequent among the Indian population [7], [28], [29], we also investigated the correlation between the HBeAg negative HIV/HBV co-infected patients and their HBV DNA levels. The results showed that about 24% of the HBeAg negative patients had undetectable HBV DNA and only 15.2% of HBeAg negative patients had a HBV DNA <2000 IU/ml, the level above which treatment is considered for HBeAg negative patients [30]. This was in contrast to the multi-national study by Thio et al [26], where about 50% of the HBeAg negative patients had HBV DNA <2000 IU/ml. Interestingly, in our study population, majority of the HBeAg negative patients had HBV DNA >2000 IU/ml (60.9%), of which 37.0% had HBV DNA ≥20,000 IU/ml (Figure 1). Hence, we indicate that the HBeAg negative HIV/HBV co-infected patients are also associated with high HBV DNA levels in our set-up and anti-HBV treatment should be considered for them as well.

Extensive use of lamivudine (3 TC), effective against both HBV (at 100 mg/day) and HIV (at 300 mg/day), as the sole anti-HBV agent in the first-line ART regimen [31] often leads to the selection of 3TC-resistant HBV strains among patients co-infected with HIV and HBV. Transmission of such strains among individuals co-infected with HIV and HBV who are treatment naive might lead to severe consequences. The major mutations associated with 3TC-resistance include the rtM204V/I primary mutation often accompanied with or without the secondary/compensatory mutations (rtL180 M and/or rtV173L) [32]. These mutations also confer cross-resistance to other anti-HBV agents and might favor the selection of vaccine-escape mutants [33], [34]. In this study, we found that two patients (3.8%) who were ART naive and were co-infected with HIV and HBV harbored both the rtM204V and rtL180 M drug resistant mutations in the HBV polymerase region. This was in accordance with other studies performed worldwide [35], . Thus, the frequency of such mutations was considerably low in our study population suggesting that pre-treatment screening for drug resistant mutations might not be required for every patient before ART initiation from this part of our country. However, sporadic screening may be useful.

Taking the above observations of this study into consideration, a strategy could be followed in order to manage the burden of HIV/HBV co-infection and for prioritizing patients according to their needs of anti-HBV therapy, mainly in the healthcare units where HBV DNA detection assays are unavailable. For subjects co-infected with HIV and HBV, the standard of care is to use two HBV active nucleoside/−tide reverse transcriptase inhibitors (NRTI/NtRTI) combination as the backbone of ART. If there is an indication to start either ART or anti-HBV treatment, the co-infected patient is usually put on tenofovir (TDF) combined with emtricitabine (FTC) or lamivudine (3 TC) plus a third agent active against HIV, as the preferred regimen [38], [39]. In our resource restricted settings, for HBeAg positive HIV/HBV co-infected subjects, ART should be initiated with combination of two such dually-active drugs without quantification of HBV DNA, owing to its distinct relationship with high HBV DNA levels. On the other hand, a similar strategy would not be that beneficial for HBeAg negative subjects. Although about 61% of HBeAg negative patients had HBV DNA ≥2000 IU/ml, significant proportion of them (p<0.0001) also had undetectable HBV DNA (Figure 1). Therefore, in such cases making treatment decisions would not be possible without HBV DNA quantification. However, if these HBeAg negative patients require ART for their clinical and/or immunological status, they should be initiated with the same dually active NRTI/NtRTI based ART regimen.

In conclusion, this study shows that the HIV infected patients are at higher risk of HBV co-infection in this cohort. Additionally, we also found that HBeAg positivity could serves as an effective marker in order to prioritize the need for anti-HBV treatment under resource-poor settings lacking the HBV DNA quantification facility. Notably, majority of the HBeAg negative HIV/HBV co-infected patients also had considerably high HBV viremia and therefore required necessary anti-HBV treatment as well. However, for HBeAg negative patients, we could not find any such surrogate markers which might help in treatment decisions, without HBV DNA quantification. In future, further work needs to be done in this regard and also to characterize HBV at the molecular level in order to elucidate the underlying elements responsible for proficiency of this infection among the HIV infected population.
